# Effects of Partial Organic Substitution for Chemical Fertilizer on Antibiotic Residues in Peri-Urban Agricultural Soil in China

**DOI:** 10.3390/antibiotics10101173

**Published:** 2021-09-27

**Authors:** Baocheng Dong, Wei Li, Wenyong Xu

**Affiliations:** 1 School of Chemical Engineering and Technology, Tianjin University, Tianjin 300350, China; nycdong@126.com; 2 Comprehensive Laboratory for Resource Recycling Technology and Model of the Ministry of Agriculture and Rural Affairs, Rural Energy and Environment Agency, Beijing 100125, China; yqxwy@163.com

**Keywords:** antibiotic contamination, soil quality, livestock manure, sewage sludge, agricultural sustainability

## Abstract

Recycling of organic wastes in agricultural ecosystems to partially substitute chemical fertilizer is recommended to improve soil productivity and alleviate environmental degradation. However, livestock manure- and sewage sludge-derived amendments are widely known to potentially carry antibiotic residues. The aim of this study is to investigate how substituting organic fertilizer for chemical fertilizer affects soil quality and antibiotic residues in agricultural soil, as well as their tradeoffs. A field experiment was conducted with the different treatments of pig manure and sewage sludge as typical organic fertilizers at equal total nitrogen application rates. The analysis of variance showed that the increments on the levels of residual antibiotics in the agricultural soils due to organic substitution for chemical fertilizer by pig manure and sewage sludge were observed. The antibiotic residues ranged from 13.73 to 76.83 ng/g for all treatments. Partial organic substitution significantly increased the sequestration of antibiotics in agricultural soil by 138.1~332.5%. Organic substitution will also significantly improve soil quality, especially for nutrient availability. Based on principal component analysis, organic substitution will strongly affected soil quality and antibiotic contamination. Pearson’s correlation showed that soil physicochemical properties had significant correlations with concentrations of antibiotics in soil, indicating organic fertilizers can promote the persistence of antibiotics in soil by modifying soil quality. To balance the benefits and risks, appropriate management practices of organic fertilizers should be adopted.

## 1. Introduction

Antibiotics have been extensively used worldwide over the last decades as drugs for preventing plant, animal, and human infections; growth promotion; and as feed additives for animals to prevent or treat diseases [1,2]. Between 2000 and 2015, global antibiotic consumption increased 65%, and the antibiotic consumption rate increased 39% [3]. Based on an estimation of antibiotic consumption, the total usage was 89,700 t in 2011 and 92,700 t in 2013 across China [4,5]. However, a significant percentage of antibiotics were excreted via urine and feces [1]. It has been widely documented that antibiotics can be detected in livestock manure and sewage sludge worldwide, even at high levels [6,7]. Antibiotics that have originated from agricultural activities, such as manure application and wastewater irrigation, can enter into agro-ecosystems [8,9]. Numerous studies have documented that exposure to antibiotics will cause serious potential risks to agro-ecosystem sustainability and human health, including toxic damage to organisms, disturbance to microbial community (in soils, animal guts, and plant phyllosphere) and induced antibiotic resistance [10,11,12,13]. Therefore, it is important to pay extra attention to antibiotic contamination of soil from agro-ecosystems.

Organic fertilizers are often applied to soil in an attempt to improve soil physicochemical and biological properties. Substituting chemical fertilizer with manure may affect crop yields, and field studies so far often focus on specific agro-environmental indicators and tend to neglect side-effects [14]. For instance, recycling of livestock manure in agro-ecosystems to partially substitute chemical fertilizer is recommended to alleviate land degradation and environmental pollution, which may also affect food security [15]. However, manure-derived amendments are known to potentially carry antibiotic residues. Numerous studies have reported the occurrence and accumulation of antibiotics in soils, especially in manure-amended agricultural soils [16,17,18]. Based on present surveys, the detectable concentrations of antibiotics in agricultural soils ranged from a few μg/kg to several mg/kg [1]. After entering soil, adsorption and desorption reactions are the notable factors that control the availability and mobility of antibiotics, which are significantly affected by soil pH, soil texture, metals ions, and organic matter [19,20]. While a number of measurements have been reported, the environmental benefits and risks of substituting chemical fertilizer with livestock manure and sewage sludge have not been systematically assessed. The effects of organic substitution for chemical fertilizers on soil quality and antibiotic contamination and their tradeoffs are still unclear. Such work is urgently needed to understand the impacts of organic substitution on the environment, as well as the underlying mechanisms. We hypothesized that organic substitution for chemical fertilizers will substantial enrich antibiotics in agricultural soil, and the improvements of soil quality caused by organic substitution will also promote persistence of antibiotics in soil.

In this study, a field experiment in typical peri-urban agricultural area is conducted to explore the effects of partial organic substitution of chemical fertilizer on soil quality and antibiotic residues. Specifically, two objectives are defined: (i) to compare the soil physicochemical properties and antibiotic concentrations in soil treated with livestock manure and sewage sludge; and (ii) to identify the correlations between soil physicochemical properties and antibiotic concentrations in organic-fertilizer-amended soil. This study is expected to improve our understanding of environmental benefits and risks caused by substituting organic fertilizer for chemical fertilizer.

## 2. Results

### 2.1. Effects of Organic Substitution on Soil Properties

The soil properties were varied with organic substitution of chemical fertilizer (Table 1). The soil clay content showed significantly higher values in the 50%P and 50%S plots than other plots, whereas the sand contents were significantly lower, which indicated that organic substitution substantially increased soil clay content and decreased the sand content. However, there were no clear differences in silt content among these treatments (*p* > 0.05).

The mean soil pH in the CK group (4.90 ± 0.27) was significantly (*p* < 0.05) lower than that of other groups with organic substitution. Inconsistently, chemical fertilizer would substantially decrease soil pH (4.44 ± 0.16). The amendment of organic fertilizers can clearly regulate the soil acidification. Moreover, soil nutrient content generally increased due to the organic substitution (*p* < 0.05). However, there were no significant differences in carbon (C), nitrogen (N), or total potassium (TK) contents between the plots with organic substitution, indicating the high substitution rate cannot substantially increase their content. However, total phosphorous (TP) content was higher in soil from the 50%S (1.70 ± 0.14 g/kg) and 50%P (1.69 ± 0.22 g/kg) treatments. The available P and K contents were also higher in 50%S and 50%P treatments. Nevertheless, the available nitrogen (AN) content was relatively higher in 100%N treatment (0.35 ± 0.04 g/kg).

### 2.2. Effects of Organic Substitution on Antibiotic Residues

**Figure 1:**
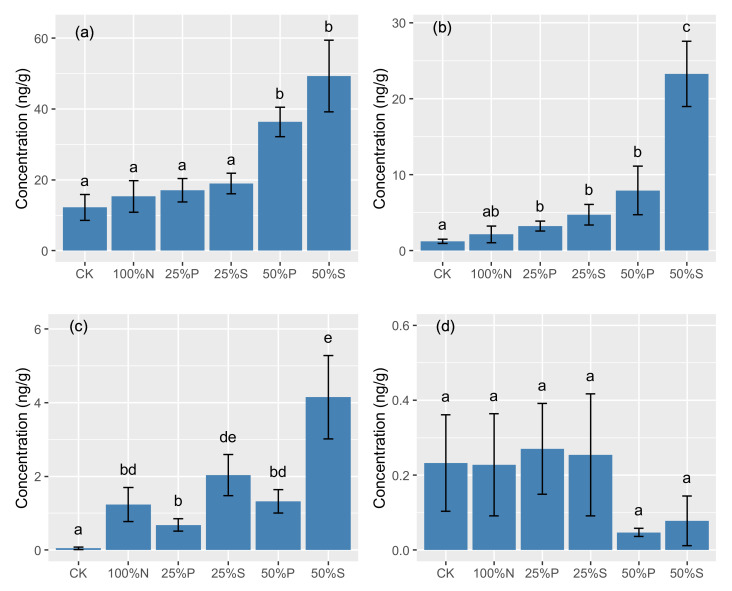
Antibiotics in soil from different treatments. (**a**) Tetracyclines, (**b**) quinolones, (**c**) sulfonamides, and (**d**) macrolides. Error bars depict standard deviations of the means. The different letters indicate significant differences among the treatments (*p* < 0.05).

In this experiment, we observed that antibiotic residues ranged from 13.73 to 76.83 ng/g for all treatments (Table 2). Compared with the 100%N treatment, PM (pig manure) and SS (sewage sludge) partial substitution significantly increased the sequestration of antibiotics in agricultural soil by 138.1%~332.5%. Moreover, on average, the 50%P and 50%S treatments produced the notable accumulation of antibiotics in soil, with concentrations of 45.65 ng/g and 76.83 ng/g, respectively. The cumulative antibiotic residues in soil over the experiment decreased in the following order: 50%S > 50%P > 25%S > 25%P > 100%N > CK. Similarly, the 50%P and 50%S treatments produced the largest accumulation of TCs in soil compared to other treatments, with average concentrations of 36.36 ng/g and 49.31 ng/g (Figure 1), whereas there were no statistically significant differences in TC (tetracycline) concentration in soil between the two organic amendment types. Notably, organic amendment will clearly increase the concentration of QNs (quinolones) in soil, and the highest concentration of QNs was from 50%S treatment (23.29 ± 11.37 ng/g), which was almost 20 times higher than CK treatment. Compared with the 100%N treatment, only 50%S treatment significantly increased the accumulation of QNs in soil. On average, both PM and SS substitution significantly increased the concentration of SAs in soil. The 50%S treatment consistently produced the largest accumulation of SAs (sulfonamides, 4.15 ± 2.99 ng/g) in soil. In contrast, no statistically significant differences in concentration of MLs (macrolides) in soil were observed between these treatments.

For the specific antibiotic compound, partial organic fertilizer substitution generally increased the residual concentration of antibiotics in soil compared with the 100%N treatment (Table 2). Sulfamethoxazole and sulfamethazine showed higher concentration in soil from 50%S treatment than other treatments, while sulfamerazine had higher concentration in 50%P treatment. Sulfamethazine was the predominant compound in SAs group, with the range of n.d. (not detection) ~3.42 ng/g. Relative to the 100%N treatment, partial substitution with organic fertilizer, especially for 50%S treatment, significantly increased the concentration of oxytetracycline, tetracycline, and doxycycline, while chlortetracycline (25.78 ng/g) showed higher concentration in soil from 50%P treatment. For QNs, 50%S treatment consistently harbored a substantially higher concentration in soil, with average concentration ranging from 0.48 ng/g to 9.24 ng/g. In contrast, MLs concentration in soil did not increase with the increasing organic substitution rate. We observed a higher concentration of erythromycin, tylosin, clarithromycin, and roxithromycin in soil from 25%P and 25%S treatments. Erythromycin had a relatively higher concentration than other MLs, especially in soil from 25%S treatment.

**Figure 2:**
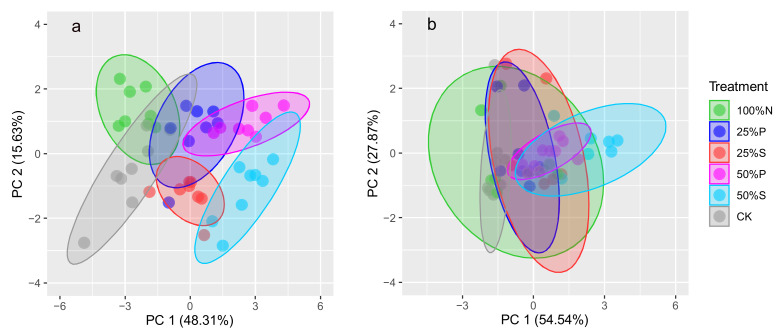
Principal component plots of (**a**) soil quality and (**b**) antibiotics with different treatments.

The PCA results are presented in Figure 2. The soil nutrients and antibiotics could be classified into various groups in organic-fertilizer-amended soil with different organic substitution rates. In case of soil quality, the clusters of CK, 100%N, 25%P, and 25%S were intersected, whereas 50%P and 50%S groups clustered with clear distance with CK group. The results suggested that 100%N treatment cannot significantly change soil quality in comparison with CK treatment, as well as 25%P and 25%S treatments. In contrast, 50%P and 50%S treatments would substantially modify soil fertility and quality. There was clear difference in soil nutrients between 25%S and 50%P treatments, which both were similar to the 50%S treatment. Moreover, most of nutrients were grouped into the first component with high explained variance (Table S1), indicating organic substitution substantially changed soil nutrients in comparison with other physicochemical properties. In case of antibiotic residues, clear distance was only observed between CK and 50%S clusters, indicating 50%S treatment significantly changed the residual concentration of antibiotics in comparison with CK treatment. Although there were no inputs of antibiotics from organic fertilizers in 100%N treatment, the antibiotic concentration and composition were similar to the treatments amended with organic fertilizers, probably due to the irrigation with antibiotic-contaminated wastewater [21]. TCs, QNs, and SAs were clustered into the first component, indicating they were the predominant antibiotics in agricultural soil amended with organic fertilizers (Table S2). However, TCs were also clustered into the second component with MLs, suggesting they were likely affected by water movement and plant accumulation [21,22].

### 2.3. Correlations between Soil Properties and Antibiotic Residues

**Figure 3:**
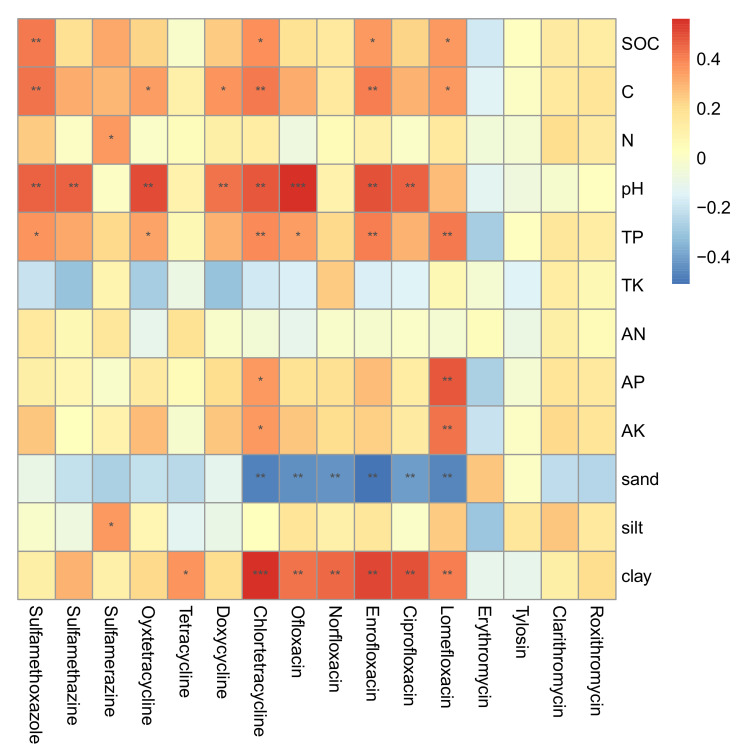
Correlations between soil properties and antibiotic residues. *: *p* < 0.05, **: *p* < 0.01, and ***: *p* < 0.001.

In general, soil properties exhibited more intensive correlations with TCs, QNs, and SAs than with MLs (Figure 3). No significant positive or negative correlations were observed between soil properties and MLs concentrations in soil from different treatments. On the contrary, significant positive correlations between soil properties and TCs, QNs, and SAs antibiotics were generally identified. For instance, soil organic and inorganic carbon contents were significantly (*p* < 0.05) and positively correlated with concentration of sulfamethoxazole, chlortetracycline, enrofloxacin, and lomefloxacin, respectively. Total carbon content also showed significantly positive correlations with concentrations of oxytetracycline and doxycycline. Nevertheless, nitrogen content only exhibited positive correlations with sulfamerazine concentration. In addition, P content also positively correlated with some antibiotics, such as lomefloxacin and chlortetracycline. Soil pH may be an important influencing factor regulating antibiotic residues. We found that the residual concentrations of most TCs, QNs, and SAs were significantly correlated with soil pH. Soil clay and sand contents were significantly and positively correlated with the concentration of QNs in soil, as well as some TCs.

## 3. Discussion

### 3.1. Organic Substitution Increases Antibiotic Concentration in Soil

In this study, we also detected antibiotics in pig-manure- and sewage-sludge-based fertilizers. Antibiotics have been heavily used in livestock; however, most of them are excreted through feces on average up to 40% [1,2]. Therefore, it is obvious that the active form of antibiotics would remain in the feces, which may remain in soil as residues. A large number of antibiotics have been found in animal manure and sewage sludge [23,24,25]. In this region, seventeen antibiotics were detected in manure-based fertilizers, and the sum concentration can be more than 15,000 ng/g [26]. The antibiotic residues enriched in organic fertilizers can enter into amended soil via ecological processes (e.g., water movement) [22]. Therefore, antibiotic concentration in soil will be substantially improved by organic amendments, and ciprofloxacin, sulfamethazine, chlortetracycline, and doxycycline are the most critical compounds in manure-amended soil [7]. We found significant increments of levels of residual antibiotics in agricultural soils due to organic substitution for chemical fertilizer, following the strong improvement of soil nutrients, including C, N, P, and K, especially for their available fractions (Table 1 and Table 2). It was also found that repeated application of manure-based organic fertilizers significantly increased antibiotic residues [9]. Similarly, Li et al. [27] observed that both dairy cattle and chicken manure significantly improved the levels of residual antibiotics. In the field environment, diverse antibiotics were widely detected in vegetable farm soils fertilized with animal manures, and TCs and QNs had the severe ecological risks [16]. Antibiotics were detected in manure-amended soils, at average concentrations ranging from 0.078 to 150 ng/g in surface layers, with the highest levels found in the fields fertilized with the swine slurry solid fraction [17]. As expected, compared with the forest sites, soils amended with pig manure presented the highest antibiotic concentrations [18,28].

Application of sewage sludge will also enrich antibiotics in agricultural soil. In China, quinolones were the dominant antibiotics detected in sewage sludge with total concentration up to 8905 ng/g [29], which was similar to the results in the United States [30]. We also detected high concentration of QNs in sewage sludge. These antibiotics can be introduced into treated soil. For example, it was noted that in secondary sludge, the highest concentrations were found for antibiotics, and the critical compounds found in the sludge-amended soil were ciprofloxacin, ofloxacin, and tetracycline [6]. Golet et al. [31] suggested sewage sludge as a main reservoir of QNs and confirmed the persistence of ciprofloxacin and norfloxacin in sludge-treated soils, although the mobility into subsoil was limited. However, none of the investigated antibiotics were detected in soils amended with digested and stored sewage sludge in southern Sweden, which indicates that long-term sludge amendment has not led to accumulation of these antibiotics in the soils [32]. In the Czech Republic, the application of sewage sludge represented a negligible risk for antibiotics in the treated soils [33]. Compared to other countries, the restricted and low use of antibiotics and/or wastewater treatment procedure can be considered as the reasons for the low antibiotic contents in the sludge-treated soils.

In this study, our results suggested that the organic substitution rate for chemical fertilizer will substantially increase antibiotic concentration in treated soil, especially for TCs and QNs (Figure 1). Previous study suggested that application rate of organic manure can be closely correlated with the ecological risks of antibiotics in soil. For instance, Ghirardini et al. [7] predicted the residual concentration of antibiotics in soil by assuming two application rates (2200 kg/ha vs. 9500 kg/ha per year) and found that there usually were relatively higher concentration of antibiotics in soil with high application rate. In contrast, the detected concentrations of antibiotics in sludge-treated soil were not obviously related to the amount of applied sludge [32]. However, antibiotic residues in soil will be influenced by numerous factors, such as climate and soil properties. At a large scale, application rate of organic fertilizer may be not positively correlated with antibiotic concentration in soil. Rahman et al. [20] did not find the higher concentration of antibiotics in soil with larger amount of manure applied on soil from different region of China. However, manure amendment will significantly lead to the enrichment of antibiotics in soil in comparison with the control and chemical fertilizer treatments [20]. In this study, organic substitution rate was the significant contributing factor to antibiotic concentrations in soil with similar environmental conditions (Figure 2).

### 3.2. The Influences of Soil Properties on Antibiotic Residues

We found clear correlations between soil properties and antibiotic concentrations (Figure 3). For example, we observed significantly positive correlations between antibiotics and carbon content (total and organic) and soil pH. This result was consistent with the previous report that antibiotic concentrations were correlated with organic carbon contents in the surface horizons with correlation coefficients higher than 0.9 for QNs [34]. Numerous studies have demonstrated that application of animal manure or sewage sludge will improve soil quality [35,36]. Nevertheless, organic amendments will also turn soil into a sink of antibiotics and antibiotic resistance [33,37]. The application of organic fertilizers improved soil physicochemical properties, which will promote the adsorption of antibiotics in soil. In the soil with long-term manure amendment, antibiotic concentrations were significantly affected by pH and organic and total carbon content [20]. The sorption capacity of tetracyclines to soils might be largely affected by cation exchange capacity and soil organic carbon, while the sorption rate, interaction strength, and equilibrium sorption binding might be affected by soil pH and soil inorganic carbon, suggesting that the predominant sorption mechanism of tetracyclines for acid soils might be hydrophobic interactions [38]. Similarly, the retention of sulfonamides was strongly influenced by the soil organic carbon content, with higher adsorption and less desorption associated with higher organic carbon content [39]. Soil pH is another important factor affecting the sorption and degradation of antibiotics, and the surface charge of soil and antibiotics depends on soil pH, which will affect the sorption and migration of antibiotics [19].

Soil texture also played important role in influencing antibiotic residues in treated soil, especially for tetracycline, chlortetracycline, and QNs (Figure 3). The chemistry of tetracyclines suggests that cation exchange with soil and sediment clay components will be their important sorption mechanisms [40]. We found that soil clay content was more important than affecting the residual concentration of tetracycline and chlortetracycline than carbon content. Our finding was consistent with previous results, which suggested the stronger adsorption of tetracycline and chlortetracycline on clays than organic matter [41]. QNs often showed high sorption to mineral/clay surfaces and to organic matter. Leal et al. [42] also confirmed that soil texture was the soil attribute that most affected sorption of QNs, and ionic exchange process seemed be an important sorption mechanism. It is expected that ciprofloxacin will have had stronger adsorption on clay minerals than oxytetracycline in soil with high cation exchange capacity [43], in line with our results. Therefore, organic substitution for chemical fertilizer in agricultural soil can enrich antibiotics via continuous loading and the modification of soil properties.

However, manure application also will neutralize or inactivate the antibiotics in soil by modifying the soil temperature and moisture. Generally, antibiotics released into soils via manure are not readily biodegradable (TCs and QNs). The mineralization accounts for less than 2% of most antibiotics in soil [1]. The photo-degradability of antibiotics in soil is an important factor in environmental fate. TCs and QNs have prolonged presence in soil, for over 100 days [1,22]. Nevertheless, warming promoted the degradation rates of soil-borne antibiotics via pH-mediated reactions such as hydrolysis and epimerization [6,7]. Application of organic fertilizers will enhance soil temperature and moisture content, and thus degradation of antibiotics.

### 3.3. Implications

Our analysis indicated that a partial substitution of N fertilizer by animal manure or sewage sludge had great potential to increase antibiotic concentration in agricultural soil. It is widely known that animal manure and sewage sludge are considered suitable for agricultural reuse, in order to maintain and/or restore soil quality and reduce the need for chemical fertilizers. Since environmental pollution is crucial for the enrichment of antibiotics in soil, several strategies should be applied in the treatment and use of organic fertilizers. Sonne et al. [44] suggested that biogas plants and biomembranes should be used to remove hazardous substances such as antibiotics before organic substitution for chemical fertilizers was used in agricultural production. Moreover, soil ecosystem sustainability and productivity will be substantially improved by dissipating manure-induced and soil-borne antibiotics [45]. For instance, admixtures (e.g., biochar) to manure can be used to lower the mobility and hence bioavailability of antibiotics [46,47].

Our experiment clearly provided new insights into the benefits and possible trade-offs of the replacement of N fertilizer by organic fertilizers. We found that organic substitution can obviously improve soil fertility. These results could be the support of the implementation of the “N Fertilizer Zero Increase Action Plan” by 2020 in China, which aims to produce more food with low environmental costs. However, partial organic fertilizer substitution should be included in decisions concerning the future of agricultural sustainability and human health. Our results suggested that compared with sewage sludge, pig manure may be a more suitable substitution of chemical fertilizer in this study area. Similarly, Tang et al. [48] comprehensively assessed the effects of substituting organic fertilizer for chemical fertilizer on net ecosystem services using a comparable economic units, and also confirmed that the substitution of pig manure had the higher economic value of ecosystem services than sewage sludge. Therefore, organic fertilizers such as livestock manure had large potential for use as fertilizers in agricultural land.

## 4. Materials and Methods

### 4.1. Site Description

The field experiment was established in Zhangxi catchment, Ningbo City, eastern China (29°47′34″N, 121°21′48″E), which is a typical peri-urban agricultural area. This region has to face the restructuring of land and population due to the rapid urbanization. To balance the demand and supply of food, the local farmers usually use a large amount of organic fertilizers to improve crop yield. The field measurements were conducted from June 2017 to July 2018. The climate of the experiment area is subtropical monsoon, with a mean air temperature of 18.1 °C and mean annual precipitation of 1382 mm. The soil in this experiment had a pH of 5.62 ± 0.15, and carbon and nitrogen content was 26.5 ± 0.08 g/kg and 3.91 ± 0.17 g/kg, respectively. Soil clay content was 28.4%, and sand content was 39.4%. The daily mean temperatures and precipitation during the observation and the main characteristics of the background soil and organic fertilizers in this study are shown in previous study [48]. The concentrations of antibiotics in pig manure and sewage sludge used in this study are shown in Table 3.

### 4.2. Experimental Design and Field Management

In this experiment, sewage sludge (SS) and pig manure (PM) were used as organic fertilizer. The substitution rate was calculated based on the N content. The field experiment consisted of the following six treatments: (1) CK (no fertilizer), (2) 100%N (100% N application using chemical fertilizer), (3) 25%S (25% of chemical fertilizer N substituted by SS), (4) 25%P (25% of chemical fertilizer N substituted by PM), (5) 50%S (50% of chemical fertilizer N substituted by SS), and (6) 50%P (50% of chemical fertilizer N substituted by PM). Each treatment had four replicates, which were randomly arranged in the field, and the plot size was 6 m × 7 m (Figure S1). Four replicate plots had different consecutive vegetable rotations using local practices, including water spinach (*Ipomoea aquatica*), spinach (*Spinacia oleracea* L.), Chinese cabbage (*Brassica rapa* L. Chinensis), and amaranth (*Amaranthus tricolor* L.). The details about the management practices in the vegetable fields were shown in Table S3, as presented in previous study [48]. All plots were constructed in the same field and had similar soil characteristics and cultivation practices. Soil samples were collected at the same time (within 3 h) by local farmers when all vegetables were harvested (15 September 2018) in each plot. Five subsamples were collected in each plot and were completely mixed. The soil samples were transported to laboratory immediately. A portion of the soil samples was gently crumbed to pass through a 2-mm sieve for soil properties analysis, and the rest were stored at −20 °C before extraction of antibiotics.

Basic soil physico-chemical properties were measured following the methods of previous study [49,50,51,52]. Soil pH was determined using an electrode-equipped pH meter with a soil-to-water ratio of 1:2.5. Soil texture was determined by the laser diffraction method using a laser particle analyzer. Total carbon (C) and total nitrogen (N) in soil were determined using an elemental analyzer. Soil organic carbon (OC) was determined using the dichromate oxidation method. Available nitrogen (AN) in soil was determined by the alkali diffusion method. Total phosphorus (TP) and total potassium (TK) were quantified by using inductively coupled plasma-atomic emission spectrometry. Available phosphorus (AP) in soil was extracted using 0.5 mol/L sodium bicarbonate, and available potassium (AK) was extracted using ammonium acetate and determined by flame photometry.

### 4.3. Analysis of Antibiotics

Four groups of antibiotic compounds, which were frequently detected in agricultural soil, were selected, including tetracyclines (TCs), quinolones (QNs), sulfonamides (SAs) and macrolides (MLs). Target antibiotic standards, including tetracycline (TC), oxytetracycline (OTC), chlortetracycline (CTC), doxycycline (DOX), ofloxacin (OFL), norfloxacin (NOR), ciprofloxacin (CIP), enrofloxacin (ENR), lomefloxacin (LOM), sulfamethoxazole (SMX), sulfamerazine (SMR), sulfamethazine (SMZ), erythromycin (ERY), tylosin (TYL), clarithromycin (CLA), and roxithromycin (ROX) were all obtained from Dr. Ehrenstorfer (Gmbh, Augsburg, Germany). Three isotope-labeled internal standards, sulfamerazine-^13^C (SMR-^13^C), tetracycline-D_6_ (TC-D_6_), and erythromycin-^13^C-D_3_ (ETM-^13^C-D_3_), were purchased from Toronto Research Chemicals (North York, ON, Canada), and another norfloxacin-D_5_ (NOR-D_5_) was purchased from Witega (Berlin, Germany).

HPLC-grade methanol and acetonitrile were obtained from Tedia Company (HPLC grade, Fairfield, OH, USA). Oasis HLB cartridges (6 mL, 200 mg or 6 mL, 500 mg) were purchased from Waters (Milford, MA, USA). Glass fiber filters (GF/F, pore size 0.7 μm) were purchased from Whatman (Maidstone, England) and kept at 450 °C for 4 h before use. Ten mg of individual standard was accurately weighed, then dissolved in 100 mL methanol and stored at −20 °C in the dark. Working solutions were obtained by gradient dilution of these stock solutions.

One gram of each soil sample was weighed into a 30 mL centrifuge tube. All samples were spiked with 20 μL of each internal standard (5.0 mg/mL). Each tube was added with 10 mL methanol-acetonitrile-acetone (*v*/*v*/*v* = 2:2:1) and 10 mL Na_2_EDTA-McIlvaine buffer (pH 4.0) [9]. The mixtures were vortexed, ultrasonicated, and then centrifuged. The procedure was repeated twice, and all the supernatants were combined, diluted to 500 mL, and then filtered using GF/F filters. The filtrate was subsequently extracted by solid-phase extraction (SPE) using HLB cartridges (Waters Oasis HLB, Milford, MA, USA). Prior to extraction, all HLB cartridges were preconditioned with 6 mL of acetone, 6 mL of methanol, and 6 mL of Milli-Q water. After the loading of filtrates, 3 mL methanol and 5 mL 1% formic acid in methanol were used to elute the target compounds from the cartridges. The extracts were purged with gentle nitrogen gas and re-dissolved to 1 mL. The final extract was transferred to a 2 mL amber vial, and then kept at −20 °C until analysis.

The high-performance liquid chromatographyetandem mass spectrometry (HPLC-MS/MS, Thermo Dionex Ultimate 3000, Waltham, MA, USA) with C18 columns (Waters Acquity UPLC BEH, 100 mm × 2.1 mm, 1.7 μm, Milford, MA, USA) was used to determine the antibiotic concentration, including tetracyclines (TCs), fluoroquinolones (FQs), sulfonamides (SAs), and macrolides (MLs). The analyses were operated in the positive electrospray ionization mode (ESI +) and multiple-reaction monitoring (MRM) mode. Quality control and quality assurance were constructed in the procedures, which were consistent with the previous study [22]. The coefficients of determination of the working calibration curve (0.2–300 ng/g) were all >0.99. The limit of detection (LOD), limit of quantification (LOQ) for antibiotics, and recovery rates are presented in Table S4 in Supplementary Materials.

### 4.4. Statistical Analysis

One-way ANOVA was used to examine the differences in the soil physicochemical properties and antibiotic residues between different treatments using the ‘plyr’ and ‘ggplot2′ packages. The *p*-values were corrected by the least significance difference (LSD) as a multiple comparisons test. Principal component analysis (PCA) was performed based on correlation matrix to show the effects of organic substitution on soil nutrients and antibiotics using ‘vegan’ package. The matrix of the weights of the PCs demonstrate the relative importance of each variable in the PC calculations. Eigenvalues higher than 1 were used to select PCs. Pearson correlation analysis was used to examine the correlation between soil properties and antibiotic residues in all plots using ‘psych’ package and visualized using the ‘pheatmap’ package [53]. All statistical tests were considered significant with a *p* value of less than 0.05. All the analysis were conducted using the packages of R 3.6.1 [54].

## 5. Conclusions

Our results demonstrate that partially substituting chemical fertilizer with organic fertilizer N had the notable potential to enrich antibiotics in soil following the improvement of soil quality. The partial substitution by organic amendments significantly increased the sequestration of antibiotics in agricultural soil by 138.1~332.5%. Moreover, residual levels of antibiotics in soil seem to be positively correlated with organic substitution rate, especially in sewage sludge-treated soil. Besides the direct loading, the persistence of antibiotics in soil can be significantly affected by soil physicochemical properties (e.g., soil pH, soil texture, and carbon content) modified by organic amendments. In these conditions, agricultural reuse of livestock manure and sewage sludge showed environmental benefits and risks. However, substitution by livestock manure was a more suitable method to balance the tradeoffs of benefits and risks in comparison with sewage sludge. The quantitative effects of replacing chemical fertilizer with livestock manure found in our study may be used for policy-making to further improve policies for substitution of chemical fertilizer by organic manure in sustainable agricultural production in China, and in other regions with similar conditions.

## Figures and Tables

**Table 1:** Soil properties in different experimental plots (mean ± standard deviation, *n* = 8).

Soil Properties	CK	100%N	25%P	25%S	50%P	50%S
Clay (%)	21.38 ± 2.20 a	25.08 ± 2.18 b	25.41 ± 1.95 b	25.00 ± 2.11 b	30.74 ± 2.36 c	29.29 ± 3.36 c
Silt (%)	37.50 ± 2.61 a	35.56 ± 3.08 a	36.80 ± 1.86 a	36.20 ± 1.91 a	37.52 ± 2.11 a	37.89 ± 2.17 a
Sand (%)	41.12 ± 2.59 a	39.37 ± 3.16 ab	37.79 ± 2.50 b	38.81 ± 1.06 b	31.74 ± 3.70 c	32.82 ± 4.74 c
pH	4.90 ± 0.27 a	4.44 ± 0.16 b	5.12 ± 0.23 a	6.65 ± 0.12 c	5.87 ± 0.09 d	7.02 ± 0.19 e
C (g/kg)	24.39 ± 2.89 a	26.17 ± 2.55 ab	32.71 ± 3.73 b	30.68 ± 5.01 b	35.79 ± 4.88 b	36.85 ± 2.74 b
N (g/kg)	3.11 ± 0.39 a	3.54 ± 0.34 ab	3.92 ± 0.56 b	3.41 ± 0.47 b	4.04 ± 0.48 b	3.61 ± 0.29 b
TP (g/kg)	0.93 ± 0.18 a	0.88 ± 0.07 a	1.44 ± 0.25 b	1.40 ± 0.22 bc	1.69 ± 0.22 d	1.70 ± 0.14 cd
TK (g/kg)	19.87 ± 0.61 a	20.01 ± 0.51 ab	20.16 ± 0.54 b	19.86 ± 0.93 b	20.37 ± 0.49 b	19.56 ± 0.71 b
OC (%)	2.38 ± 0.31 a	2.51 ± 0.23 ab	3.07 ± 0.43 b	2.89 ± 0.41 bc	3.48 ± 0.46 c	3.32 ± 0.32 c
AN (g/kg)	0.25 ± 0.03 a	0.35 ± 0.04 b	0.31 ± 0.04 d	0.26 ± 0.03 ac	0.30 ± 0.04 cd	0.29 ± 0.04 cd
AP (mg/kg)	118.30 ± 24.06 a	126.86 ± 11.67 a	208.30 ± 25.16 b	159.68 ± 11.58 c	267.13 ± 27.67 c	190.63 ± 15.63 d
AK (mg/kg)	407.40 ± 32.36 ab	390.99 ± 24.20 a	433.73 ± 26.00 a	402.92 ± 22.03 b	486.32 ± 17.13 b	457.13 ± 28.82 c

Note: C: total carbon content; N: total nitrogen content; TP: total phosphorous content; TK: total potassium content; OC: organic carbon content; AN: available nitrogen content; AP: available phosphorous content; AK available potassium content. (**1**) CK (no fertilizer), (**2**) 100%N (100% N application using chemical fertilizer), (**3**) 25%S (25% of chemical fertilizer N substituted by sewage sludge), (**4**) 25%P (25% of chemical fertilizer N substituted by pig manure), (**5**) 50%S (50% of chemical fertilizer N substituted by sewage sludge), and (**6**) 50%P (50% of chemical fertilizer N substituted by pig manure). Different letters indicate significant differences among the treatments at 0.05 level.

**Table 2:** Antibiotic residues in soil from different treatments (mean ± standard deviation, ng/g, and dry weight).

Antibiotics		CK	100%N	25%P	25%S	50%P	50%S
SAs		0.04 ± 0.09	1.23 ± 1.22	0.68 ± 0.44	2.03 ± 1.47	1.32 ± 0.83	4.15 ± 2.99
	SMX	0.03 ± 0.06	0.16 ± 0.24	0.22 ± 0.52	0.47 ± 0.19	0.17 ± 0.67	0.71 ± 0.11
	SMZ	n.d.	1.05 ± 1.27	0.45 ± 1.74	1.55 ± 0.39	1.13 ± 3.23	3.42 ± 0.80
	SMR	0.02 ± 0.04	0.01 ± 0.02	0.01 ± 0.02	0.02 ± 0.02	0.03 ± 0.03	0.02 ± 0.06
TCs		12.2 ± 9.75	15.3 ± 11.7	17.1 ± 8.70	18.9 ± 7.73	36.3 ± 10.9	49.3 ± 26.8
	OTC	0.26 ± 0.56	0.5 ± 1.00	1.07 ± 1.80	0.97 ± 0.96	2.1 ± 8.59	10.92 ± 1.43
	TC	1.12 ± 1.28	3.51 ± 3.63	3.55 ± 3.57	2.88 ± 5.60	3.43 ± 2.46	3.71 ± 0.67
	DOX	1.43 ± 0.98	1.98 ± 2.06	3.85 ± 2.92	2.83 ± 3.54	5.04 ± 10.9	10.93 ± 2.48
	CTC	9.4 ± 7.90	9.36 ± 9.00	8.64 ± 4.45	12.29 ± 2.49	25.78 ± 11.5	23.75 ± 8.81
QNs		1.23 ± 0.76	2.15 ± 2.92	3.25 ± 1.75	4.73 ± 3.61	7.92 ± 8.44	23.2 ± 11.3
	OFL	0.22 ± 0.21	0.18 ± 0.40	0.5 ± 2.09	1.91 ± 0.32	2.5 ± 5.54	9.24 ± 4.53
	NOR	0.21 ± 0.27	0.28 ± 0.30	0.29 ± 0.25	0.19 ± 0.37	0.45 ± 0.30	0.48 ± 0.39
	ENR	0.27 ± 0.20	0.39 ± 0.33	1.19 ± 0.88	1.2 ± 0.59	2.39 ± 4.91	6.28 ± 1.93
	CIP	0.33 ± 0.38	1.28 ± 2.71	0.62 ± 1.17	1.3 ± 0.83	1.8 ± 3.67	6.34 ± 2.19
	LOM	0.21 ± 0.39	0.02 ± 0.04	0.65 ± 0.18	0.13 ± 0.67	0.79 ± 0.39	0.95 ± 0.87
MLs		0.23 ± 0.34	0.22 ± 0.36	0.26 ± 0.32	0.25 ± 0.43	0.04 ± 0.02	0.07 ± 0.17
	ERY	0.15 ± 0.34	0.22 ± 0.36	0.17 ± 0.40	0.23 ± 0.29	n.d.	n.d.
	TYL	0.04 ± 0.05	n.d.	0.02 ± 0.03	0.02 ± 0.03	n.d.	0.02 ± 0.01
	CLA	0.02 ± 0.01	0.01 ± 0.01	0.03 ± 0.01	n.d.	0.02 ± 0.04	0.02 ± 0.01
	ROX	0.02 ± 0.02	0.01 ± 0.01	0.05 ± 0.01	0.01 ± 0.07	0.02 ± 0.08	0.04 ± 0.02
Total		13.73 ± 9.62	18.96 ± 12.6	21.31 ± 8.28	25.99 ± 7.41	45.65 ± 15.7	76.83 ± 35.0

Note: tetracyclines (TCs), quinolones (QNs), sulfonamides (SAs) and macrolides (MLs). Target antibiotics include tetracycline (TC), oxytetracycline (OTC), chlortetracycline (CTC), doxycycline (DOX), ofloxacin (OFL), norfloxacin (NOR), ciprofloxacin (CIP), enrofloxacin (ENR), lomefloxacin (LOM), sulfamethoxazole (SMX), sulfamerazine (SMR), sulfamethazine (SMZ), erythromycin (ERY), tylosin (TYL), clarithromycin (CLA), and roxithromycin (ROX). n.d. indicates not detected.

**Table 3:** Antibiotics in pig manure and sewage sludge (ng/g, dry weight).

Antibiotics	Pig Manure		Sewage Sludge	
	Mean	Standard Deviation	Mean	Standard Deviation
Oxytetracycline	1216.02	658.40	222.67	89.61
Chlortetracycline	19,820.02	7898.04	235.70	39.81
Tetracycline	3966.35	1758.68	82.65	50.11
Doxycycline	1348.94	747.90	1396.94	614.97
Norfloxacin	73.12	88.41	170.90	58.34
Ofloxacin	96.61	103.22	174.28	89.52
Ciprofloxacin	59.15	77.45	4610.81	2209.96
Lomefloxacin	0.50	0.97	0.33	0.42
Enrofloxacin	69.79	57.69	37.47	23.05
Sulfamethoxazole	202.33	212.70	29.79	18.64
Sulfamethazine	6069.81	6381.04	12,907.08	4840.82
Sulfamerazine	198.32	87.93	518.50	252.29
Erythromycin	2.10	6.31	0.11	0.18
Tylosin	29.61	30.70	56.20	32.93
Clarithromycin	0.32	0.55	2.76	2.02
Roxithromycin	0.08	0.14	4.95	3.32

## Data Availability

Not applicable.

## Supplementary Materials

The following are available online https://www.mdpi.com/article/10.3390/antibiotics10101173/s1, Table S1: Matrix of loading scores for the principal component analysis of soil quality, Table S2: Matrix of loading scores for the principal component analysis of antibiotic residues, Table S3: Cultivation and fertilization management practices in the vegetable field, Table S4: Recoveries, limits of detection (LOD) and limits of quantification (LOQs) for the determining method in soil, Figure S1: Sketch map of experimental blocks in this study.
