# Supplementary material for: Effects of Partial Organic Substitution for Chemical Fertilizer on Antibiotic Residues in Peri-Urban Agricultural Soil in China
Source: Antibiotics (Basel). 2021 Sep 27;10(10):1173. doi: 10.3390/antibiotics10101173 (PMC8532921; doi:10.3390/antibiotics10101173)
Supplement: Supplementary file 1 [file antibiotics-10-01173-s001.zip › antibiotics-1353835-supplementary.pdf]

Table S1. Matrix of loading scores for the principal component analysis of soil quality.

Variables	PC1	PC2	PC3	PC4
SOC	0.38	0.11	-0.25	0.08
pH	0.30	-0.47	0.10	0.09
C/N	0.33	-0.43	0.04	0.14
TP	0.40	-0.02	-0.11	0.11
TK	0.00	0.42	0.68	-0.16
AN	0.07	0.52	-0.59	0.10
AP	0.37	0.18	0.11	0.19
AK	0.34	0.20	0.13	0.14
sand	-0.34	-0.10	-0.05	0.47
silt	0.13	-0.12	-0.21	-0.81
clay	0.32	0.18	0.18	-0.01
Eigenvalue	5.31	1.72	1.17	1.15
Explained variation (%)	48.31	15.63	10.59	10.47

Table S2. Matrix of loading scores for the principal component analysis of antibiotic residues.

Variables	PC1	PC2
SAs	0.58	0.06
TCs	0.48	0.53
QNs	0.61	-0.13
MLs	-0.26	0.84
Eigenvalue	2.18	1.11
Explained variation (%)	54.54	27.87

Table S3. Cultivation and fertilization management practices in the vegetable field [48].

Vegetable	Management Date			Nutrient Input (kg/ha)		
	Nutrient Input	Harvest Time	Herbicide	N	P_2O_5	K_2O
Water spinach	2017/7/1	2017/7/22	2017/6/22	240	240	240
	2017/8/15	2017/9/6		180	180	180
Spinach	2017/10/29	2018/1/13	2017/10/19	180	100	180
Chinese cabbage	2018/3/15	2018/5/1	2018/3/5	180	68.58	120
Amaranth	2018/5/9	2018/6/20	2018/5/5	135	68.58	120

Table S4. Recoveries, limits of detection (LOD) and limits of quantification (LOQs) for the determining method in soil.

Groups	Analytes	Internal Standard	LODs (ng/g)	LODs (ng/g)	Recovery ± SD (%)
SAs	Sulfamerazine	SMR-^{13}C	0.1	0.3	71.6±13.8
	Sulfamethazine	SMR-^{13}C	0.05	0.2	50.3±11.1
	Sulfamethoxazole	SMR-^{13}C	0.1	0.3	84.4±14.2
TCs	Chlortetracycline	TC-D_6	2	6.7	88.9±14.6
	Oxytetracycline	TC-D_6	2	6.7	75.2±15.0
	Tetracycline	TC-D_6	2	6.7	80.6±12.8
	Doxycycline	TC-D_6	2	6.7	104.8±14.9
QNs	Norfloxacin	NOR-D_5	0.5	1.7	96.2±15.2
	Ciprofloxacin	NOR-D_5	0.5	1.7	65.3±13.8
	Enrofloxacin	NOR-D_5	0.1	0.3	59.6±12.3
	Ofloxacin	NOR-D_5	0.2	0.7	71.3±14.0
	Lomefloxacin	NOR-D_5	0.2	0.7	106.9±14.6

MLs	Erythromycin	ERY-^{13}C-D_3	0.05	0.2	90.0±13.9
	Clarithromycin	ERY-^{13}C-D_3	0.01	0.03	93.4±19.1
	Tylosin	ERY-^{13}C-D_3	0.05	0.2	70.5±13.9
	Roxithromycin	ERY-^{13}C-D_3	0.5	1.7	94.6±9.3

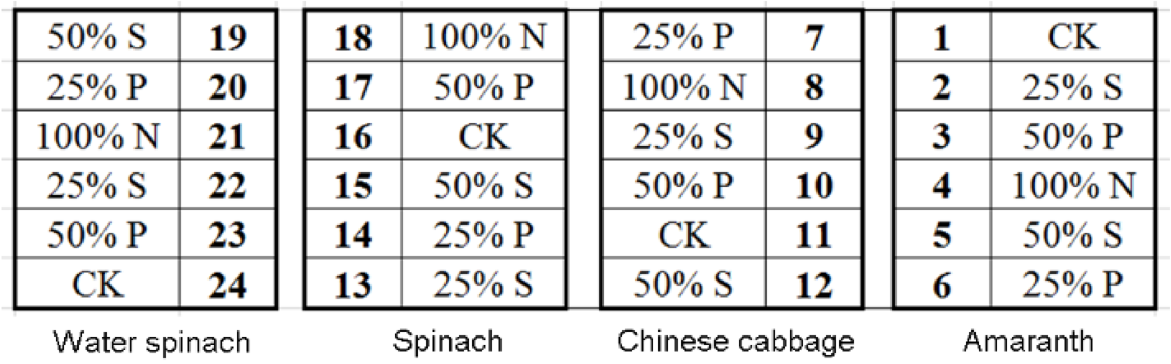

Figure S1. Sketch map of experimental blocks in this study.

References:

[48] Tang, Q.; Ti, C.; Xia, L.; Xia, Y.; Wei, Z.; Yan, X. Ecosystem services of partial organic substitution for chemical fertilizer in a peri-urban zone in China. *J. Clean. Prod.* **2019**, 224, 779-788.
